# Noncoding RNAs Emerging as Drugs or Drug Targets: Their Chemical Modification, Bio-Conjugation and Intracellular Regulation

**DOI:** 10.3390/molecules27196717

**Published:** 2022-10-09

**Authors:** Jin Wang, Tian Tian, Xin Li, Yan Zhang

**Affiliations:** 1 State Key Laboratory of Analytical Chemistry for Life Sciences, Jiangsu Key Laboratory of Advanced Organic Materials, School of Chemistry and Chemical Engineering, Chemistry and Biomedicine Innovation Center (ChemBIC), Nanjing University, Nanjing 210023, China; 2 New Drug Screening Center, Jiangsu Center for Pharmacodynamics Research and Evaluation, China Pharmaceutical University, Nanjing 210009, China

**Keywords:** noncoding RNA, therapeutics, chemical modification, RNA regulating molecules, RIBOTAC

## Abstract

With the increasing understanding of various disease-related noncoding RNAs, ncRNAs are emerging as novel drugs and drug targets. Nucleic acid drugs based on different types of noncoding RNAs have been designed and tested. Chemical modification has been applied to noncoding RNAs such as siRNA or miRNA to increase the resistance to degradation with minimum influence on their biological function. Chemical biological methods have also been developed to regulate relevant noncoding RNAs in the occurrence of various diseases. New strategies such as designing ribonuclease targeting chimeras to degrade endogenous noncoding RNAs are emerging as promising approaches to regulate gene expressions, serving as next-generation drugs. This review summarized the current state of noncoding RNA-based theranostics, major chemical modifications of noncoding RNAs to develop nucleic acid drugs, conjugation of RNA with different functional biomolecules as well as design and screening of potential molecules to regulate the expression or activity of endogenous noncoding RNAs for drug development. Finally, strategies of improving the delivery of noncoding RNAs are discussed.

## 1. Introduction

In the gene expression process, RNA is at a central position between DNA and protein, which is the product of DNA transcription and also serves as the blueprint for the translation of proteins. However, only less than 2% of RNAs can be translated into proteins [1], while the remaining 98% without coding potential are known as noncoding RNAs (ncRNAs). ncRNAs were initially considered as “evolutionary junk” with no biological function. However, in the past decades, accumulating evidences have suggested that various types of ncRNAs are involved in many important cellular processes [2].

**Figure 1:**
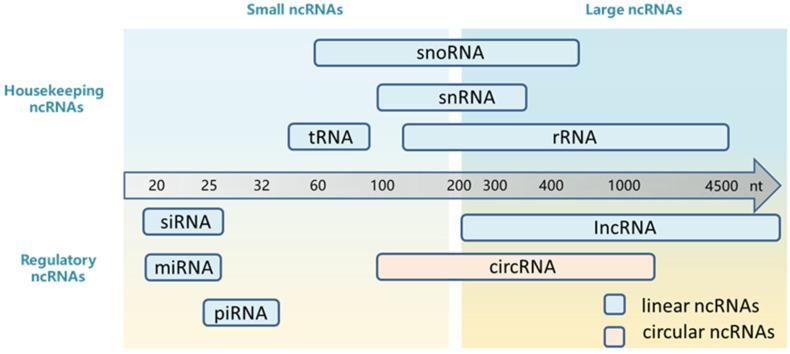
Classification and molecular size of ncRNAs.

According to the different regulatory roles, ncRNA transcripts have been categorized into two families: housekeeping ncRNAs and regulatory ncRNAs. Housekeeping ncRNAs contain transfer RNA (tRNAs) for carrying amino acids, ribosomal RNAs (rRNAs) for mRNA reading and decoding, small nuclear RNAs (snRNAs) for RNA splicing and small nucleolar RNAs (snoRNAs) for RNA modification. Another family of ncRNAs is regulatory ncRNAs, which are regulators of gene expressions at epigenetic, transcriptional, and post-transcriptional levels. Regulatory ncRNAs consist of microRNAs (miRNAs), small interfering RNAs (siRNAs), long noncoding RNAs (lncRNAs), circular RNAs (circRNAs) and PIWI-interacting RNAs (piRNAs). Based on the molecular length, regulatory ncRNAs were generally classified into two subclasses: long (or large) ncRNAs (>200 nucleotides) (nt) and short (or small) ncRNAs (<200 nt). Linear long noncoding RNAs (lncRNAs) and circular RNAs (circRNAs) are distinguished by their shapes. Small ncRNAs (sncRNAs) include microRNAs (miRNAs), small interfering RNAs (siRNAs) and PIWI-interacting RNAs (piRNAs) (Figure 1) [3].

ncRNAs play important regulatory roles in physiological processes. The interaction between ncRNA and target sequences leads to significant effects in different cellular programs, such as RNA maturation, RNA processing, signaling, gene expression, and protein synthesis [2]. On the other hand, aberrantly expressed ncRNAs are closely related with the pathogenesis of various diseases such as cancers. Under complex cancer conditions, the deregulation or dysfunction of ncRNAs has been reported to affect multiple cellular processes in almost all major cancers [4,5,6,7]. The roles of cancer-involved ncRNAs such as miRNA, lncRNA and circRNA have been extensively studied, and their potential mechanisms in cancer development are progressively being established [8,9]. Although the majority of miRNAs are still to be discovered, many of them have been demonstrated to link to cancers as oncogenes or tumor suppressors, or metastatic miRNAs [7]. Similarly, lncRNAs have been found to work in oncogenic or tumor suppressor manners as well [10]. Some circRNAs [11] and piRNAs [12] have also been identified to play an important role in the progress of cancers.

ncRNA-based drugs, which refer to ncRNAs or their derivatives used as drugs, can interact with multiple biological targets (e.g., nucleic acids and proteins) within cells and exert therapeutic abilities. As the effect of ncRNAs in various diseases has been increasingly revealed, it is possible to design novel ncRNA-based drugs for clinic use. ncRNA-based drugs, different from the current pharmacotherapy such as small molecule drugs or antibody drugs, offer a brand new perspective in drug design. The simple structures, the specific complementary binding mode and the post-transcriptional regulation made ncRNAs attractive therapeutical molecules. ncRNA molecules could be endogenous or exogenous, natural or artificial modified, which endow ncRNAs different roles in disease theranostics.

## 2. Noncoding RNAs with Theranostic Applications

The emerging links between noncoding RNAs and diseases have provided opportunities not only for understanding the prognostic mechanism, but also for finding novel diagnostic biomarkers.

Many miRNAs, particularly circulating miRNAs in serum, plasma, urine, and saliva, have already successfully served as biomarkers for many different diseases. In the context of cancer, the oncogenes (or “onco-miRs”) such as miR-126, miR-17-92 cluster, miR-210 and miR-21 are highly expressed in cancer cells and promote cancer development [13,14,15,16]. Conversely, miRNAs such as miR-34a, let-7, miR-200 and miR-122 are downregulated in cancers as tumor suppressors [17,18,19,20]. The dynamic balance between fine regulation of oncogenes and tumor suppressors may indicate the early stage of tumoral development [21]. Moreover, many ncRNAs are significant factors in diseases including cardiovascular diseases, metabolism disease, infectious diseases, immune disorders and neurological diseases [10,11,12,21,22]. Differential expression levels of ncRNAs under disease conditions make them potential biomarkers for diagnose.

For instance, increased levels of miR-21 have been reported as clinical diagnostic and prognostic marker in various cancers. The serum levels of circulating miR-122, miR-22, and miR-34a were correlated with liver injury in HIV patients and may serve as biomarkers [23]. After the outbreak of COVID-19, dozens of studies have shown that dysregulation of certain miRNAs in the COVID-19 patients has a critical impact in terms of viral activity, host responses, severity and mortality [24], suggesting that miRNAs may also serve as potential predictors for COVID-19 [22,24,25]. Recently, emerging evidences have shown that one kind of tRNA-derived RNAs (tsRNAs) was reported as diagnostic biomarkers in several diseases including epilepsy, clear cell renal cell carcinoma and gastric cancer [26]. In addition, lncRNAs, circRNAs and piRNAs [26,27] have also been used as biomarkers for diagnosis.

On the other hand, some endogenic ncRNAs control the expression of disease-related genes. The aberrant expression of these ncRNAs induces the emergence of diseases, making these ncRNAs potential targets for drug development. Aside from disease-associated miRNAs and lncRNAs, intron–exon junctions and repetitive RNAs are also considered as therapeutic targets for drug design [28]. Therefore, manipulation of their expressions could be used as a new clinical treatment strategy.

The agents used for targeting ncRNAs can be either small molecules or synthetic oligonucleotides. Traditional small molecules targeting specific RNAs are still promising as a therapeutic strategy for diseases. However, due to the similarity of intracellular nucleic acid structures, it is challenging to find small molecules that can both selectively bind the target and be specifically active to the target. Therefore, it is crucial to screen small molecules against specific ncRNAs as drugs. Early this year, Aguilar et al. established a screening strategy that can identify ncRNA binding drug-like compounds with functional effects on phenotype. With this strategy, the RNA-targeted compounds with disruption in activity can be systematically screened [29].

Alternatively, it is much easier to design an oligonucleotide than a small molecule. The complementary base-pairing recognition makes some “undruggable” targets for small molecules accessible. There are several classes of oligonucleotides for targeting ncRNAs. The most common strategies are antisense oligonucleotides (ASOs) and duplex RNAs that elicit RNA interference. Other oligonucleotides-based agents, such as ribozyme and CRISPR-cas13, could target and degrade the ncRNA in cells.

## 3. Noncoding RNAs in Approved and Emerging Nucleic Acid Drugs

Nucleic acid therapeutics use synthetic oligonucleotides to modulate gene expressions for therapeutic effects [30]. Since 1970s, nucleic acid therapeutics have drawn extensive attention [31]. The thriving studies in introducing nucleic acid molecules into cells to permanently or transiently modulate the disease related genes paved a promising new way in treating diseases. Aside from small molecules and antibody drugs, nucleic acid drugs, particularly oligonucleotide-based drugs are now considered as the third major drug discovery platform. To date, there are 16 nucleic acid therapeutics approved by the United States Food and Drug Administration (FDA) and the European Medicines Agency (EMA), most of which target orphan genetic diseases (Table 1).

**Figure 2:**
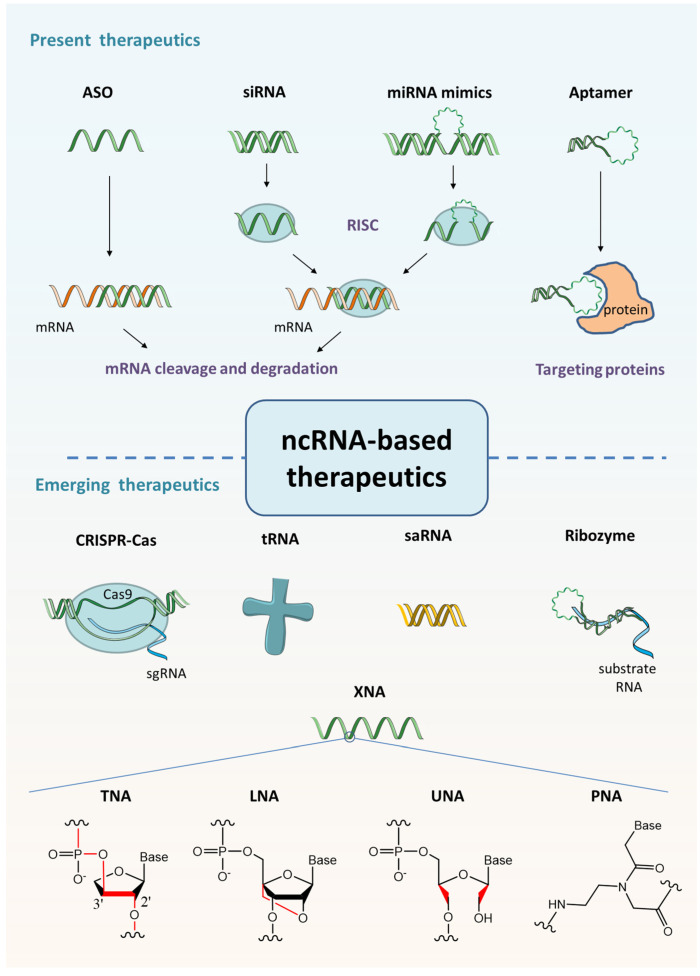
The structure and function of approved and emerging ncRNA-based nucleic acid drugs.

The discovery of ncRNAs of different structures and functions has promoted the mechanistic understanding and possible therapeutic interventions. There are several strategies to design ncRNA theraputics, such as inhibiting RNA activity by siRNA or antisense RNAs, targeting proteins by aptamers, reprogramming genetic information by trans-splicing ribozyme, targeting promoter sequences by saRNA to trigger gene production, gene modifying by CRISPR guide RNAs (Figure 2). These types of nucleic acid drugs and some other emerging ncRNA-based agents will be introduced below.

Short interfering RNA (siRNA) is a class of double-stranded RNA with 20–25 nt, which triggers the RNA interference (RNAi) pathway in mammalian cells. siRNAs are used as gene silencing tools for investigating gene function and as nucleic acid drugs [32,33]. To date, five siRNA drugs have been used in clinic. In 2018, the first siRNA drug Onpattro (patisiran) was approved by FDA for treating hereditary transthyretin-mediated (hATTR) amyloidosis [34]. Aside from rare diseases, siRNA drug Leqvio is used to lower the levels of low-density lipoprotein (LDL) cholesterol. Several siRNAs are under clinical trials to treat different pathologies.

MicroRNAs (miRNAs) are evolutionary conserved, endogenous single-stranded, small ncRNAs, influencing target mRNA expression via RNAi [35]. miRNAs play fundamental functional roles in regulating gene expression at a post-transcriptional level. To alter miRNA levels, there are two approaches: (1) miRNA mimics (miRNA-like dsRNAs) to boost miRNA expression and (2) antagomirs (anti-miRNAs) with complementary sequences to miRNAs to downregulate the target miRNAs level. Several miRNAs are under clinical trials and have shown positive results in the initial phases. However, some miRNA drug candidates have been tested to exert severe adverse effects. Further improvements such as the delivery of specifically localized miRNAs to selected target sites are needed to get the clinical approval.

RNA activation (RNAa), unlike RNAi pathway induced by miRNAs and siRNAs, is a process where dsRNA triggers gene production by targeting promoter sequences. Small activating RNA (saRNA) is a class of small dsRNA oligonucleotides synthesized by homologous sequences close to or within gene promoters, which can positively and reversibly upregulate the target genes by RNA-induced transcriptional activation (RITA) complex in an Ago2-dependent manner [36,37,38]. MTL-CEBPA is the first saRNA in clinical trials. It induces increased expression of a tumor suppressor CCAAT/enhancer-binding protein alpha (CEBPα). It is presently in Phase I clinical trials for hepatocellular carcinoma (HCC) [39].

ASOs are short, single-stranded RNA or DNA oligonucleotides that bind complementarily to the target mRNA. ASO drugs usually follow three types of mechanisms: (1) recruiting RNase H and promoting degradation of mRNA, (2) sterically blocking the interaction between mRNA and ribosome, (3) alternative splicing of pre-mRNA in exon skipping or exon inclusion manner [40,41]. By now, nine ASO drugs have been approved for clinical applications, most of which target rare disease such as Duchenne Muscular Dystrophy (DMD). The high-throughput ASO screening platforms and AI technology promote the design of ASO sequences with high efficacy, low off-target toxicity and clear mode of action. The main concern of ASOs is how to maintain the structural integrity and enhance the absorption during the delivery [42].

Aptamers are single-strand oligonucleotides with tertiary structure that can bind and modulate specific proteins. The screening system known as systemic evolution of ligands by exponential enrichment (SELEX) is used to produce aptamers in vitro. Aptamers are called chemical antibodies because the synthetic molecules act similarly to antibodies [43]. Pegaptanib (Macugen) is a 28-nucleotides RNA aptamer targeting VEGF. It is the first and only FDA-approval therapeutic aptamer to treat age-related macular degeneration (AMD) [44]. There are several aptamer drugs in clinical trial towards different types of tumors and other diseases. Aptamers have also been applied to inhibit SARS virus and the diagnosis of COVID-19 by targeting antigenic viral proteins [45].

Ribozymes are RNA molecules with enzymatic activity. They bind to the target RNA and lead to RNA cleavage in a recycling manner [46] Ribozymes are composed of two domains: the recognizing region for target binding and the catalytic domain [47]. Ribozymes require a conserved target gene, which plays critical part in biological processes and accessible sterically for a smooth binding. Hence, ribozyme has relatively higher specificity and lower immunogenicity than some other RNA drugs. The simple structure, negligible toxicity, site-specific cleavage activity and recyclable catalytic potential of ribozymes benefit their effective modulation of gene expression. Several ribozymes have already been tested in clinical trials for solid tumors, HIV, SARS and other disease [45,47]. However, it is challenging due to the new mutations in the conserved target region and RNase degradation.

The rise of CRISPR (Clustered Regularly Interspaced Short Palindromic Repeats)-Cas (CRISPR-associated gene) systems has significantly improved the performance of genome editing. The CRISPR system is composed of (1) a noncoding RNA known as guide RNA (gRNA) for targeting the sequence to be engineered, (2) the endonuclease Cas to introduce site-specific breaks in DNA or RNA [48]. The CRISPR-Cas9 system as a DNA editing tool has been broadly used in genome modification for biology and therapeutic applications. In addition, a RNA editing CRISPR-Cas system, CRISPR-Cas13, has also been developed, which has a higher safety profile than CRISPR-Cas9 [47]. Recently, the CRISPR-Cas13-based system named PAC-MAN (prophylactic antiviral CRISPR in human cells) has been used in the treatment of SARS-CoV-2 [45]. More advanced RNA editing platforms that can elicit ADAR-mediated RNA editing, have been engineered in humans, namely CIRTS, RESCUE, RESTORE and LEAPER [49].

Xeno nucleic acids (XNA) are artificial nucleic acids with chemical modifications on the natural sugar backbone of DNA and RNA [50]. XNA such as locked nucleic acid (LNA), unlocked nucleic acid (UNA), threose nucleic acids (TNA) and peptide nucleic acids (PNA) were used as therapeutic agents or tools. Normally, XNA possesses superior resistance to nuclease degradation and thus displays high biological stability. Similar to natural RNA, XNA could fold into tertiary structures and performed RNA-like biological functions, such as binding towards complementary mRNA, suppression of target gene as an antisense agents and inhibition of tumor growth in a xenograft model. Recent researches illustrated that two TNA sequences screened by in vitro selection possess RNA ligase activity and ribozyme-like RNA endonuclease activity, respectively. These findings provided TNA as an attractive XNA-based molecular tool for future biomedical applications [51,52].

Very recently, several biotechnology companies including Alltrna, ReCode Therapeutics, Shape Therapeutics, Tevard Biosciences and hC Bioscience started the pursuit of of tRNA-based therapeutics. The companies are focused on designing tRNAs to bypass premature termination codons and incorporate desired amino acids instead. The premature termination codons, which cause truncation of translated protein, are responsible for an estimated 11% of all inherited disease. Suppressor tRNAs (sup-tRNAs) can read through a premature stop codon to restore production of full-length proteins. It may open the door for an entire new class of tRNA therapies [53].

## 4. Chemical Modifications of Noncoding RNA Backbone for Drug Development

Traditional small molecule and monoclonal antibody drugs require the recognition of complex spatial conformations of target proteins, but many disease-associated target proteins, whose surface lack some specific hydrophobic pockets, cannot be screened for highly active and high-affinity drug molecules. Thus, these diseases are considered to be unavailable for drug discovery. In contrast, noncoding RNAs like miRNA or siRNA are simpler to design and can theoretically be used to down-regulate the expression of almost all the genes in the body through Watson-Crick base pairing with target mRNAs, offering a broader therapeutic scope than small molecule drugs [54,55]. Despite the great clinical potential of noncoding RNAs, they are susceptible to degradation by endogenous enzymes or rapid renal clearance in the human body, and have poor stability and pharmacokinetics. At the same time, RNA is negatively charged and has a large molecular weight, making it difficult to penetrate the membrane into the cell. These problems have hindered the rapid development of nucleic acid drug technology [56,57,58,59]. Since the development of antisense therapies in the 1980s, some chemical modification methods of RNA have emerged. Precise modification of noncoding RNAs can improve their efficacy, specificity and stability, and reduce their toxicity and immunogenicity. Currently, the mainstream modification methods are: (i) ribose modification; (ii) base modification; (iii) phosphate backbone modification [60].

As the siRNA and miRNA gene silencing activity is not dependent on ribose 2′-OH, the chemical modification of 2′-OH has little effect on biological function of small nucleic acid drugs. Currently, the dominant modification strategy is to replace 2′-OH with other chemical groups, including 2′-O-methyl (2′-O-Me), 2′-fluoro (2′-F), 2′-O-methoxyethyl (2′-O-MOE), etc. Modification of ribose 2′-OH can significantly enhance the ability of RNA to resist nucleases, while being able to reduce RNA-mediated immunogenic responses [61,62,63]. The anti-miRNA oligonucleotides (AMO) introduced into the cell have the ability to degrade or form random duplexes with specific endogenous miRNAs. 2′-O-methyl (2′-OMe) RNA-modified AMO has higher binding affinity to RNA targets and higher resistance to nucleases [64]. In addition to 2′-C, 4′-C modification, even the entire sugar ring can be modified. Locked nucleic acid (LNA) is a bicyclic structure containing a methylene bridge between 2′-O and 4′-C to produce a stable ‘locked’ ring conformation, thus increasing the resistance to nuclease degradation and significantly enhancing base pairing affinity [65]. Unlocked nucleic acid (UNA) ribose ring has higher flexibility and thermal stability due to the lack of chemical bond between C2′ and C3′. In addition, UNA modification in the seed region of the guide strand can reduce the off-target effects of miRNAs and has potential to develop novel therapeutic miRNAs [66].

Base modification of NTPs is a common method for introducing additional functionality and increasing chemoselectivity. C5′ of the pyrimidine ring and C2′ of the purine ring are common modification sites. Substitution of base analogues such as pseudouridine, 2-thiouridine, N6-methyladenosine (m6A) and 5-methylcytidine can enhance the stability of RNA while reducing its innate immunogenicity. It has been shown that m6A promotes primary miRNA processing and X-inactive specific transcript (XIST)-mediated transcriptional repression [62]. Zhang et al. found that the introduction of a 5-nitroindole-modified nucleotide at position 15 of siRNA sense strand could greatly reduce its activity without affecting the biological function of the antisense strand. This modification provides a practical strategy to reduce off-target effects mediated by sense strand [67]. Besides, 6′-phenylpyrrolidine (PhpC) is a cytosine mimetic with excellent base-pairing fidelity, thermal stability and high fluorescence. PhpC-containing siRNAs show gene silencing activity similar to that of the parent molecule, and their fluorescent properties render them applicability in fluorescence-based assays and the exploration of cellular uptake as well as transport of siRNAs [68].

Phosphate backbone modifications improve the stability of nucleic acids primarily by replacing the phosphodiester bond with other types of bonds. Phosphorothioate (PS) is the most widespread strategy and this modification primarily uses the replacement of a non-bridging oxygen of phosphodiester by a sulphur atom. PS-modified nucleotides are more resistant to nucleases and are able to prolong the half-life of drug. PS was first applied in the modification of antisense oligonucleotides (ASO). Although the modification of siRNA could enhance the stability, it is also accompanied by the side effect of increased toxicity and reduced gene silencing. This may be because siRNAs only tolerate limited modifications to remain RNA-induced silencing complex (RISC) compatible. In addition to PS, other residues have been also successfully used to replace phosphodiester groups in oligonucleotides, including dithiophosphate (PS2), methyl phosphate (MP), methoxypropyl phosphate (MOP), and peptide nucleic acid (PNA). Although less popular than PS, these modifications have the same potential for clinical application development [69,70,71].

## 5. Bioconjugation of Noncoding RNA with Other Biomolecules

**Figure 3:**
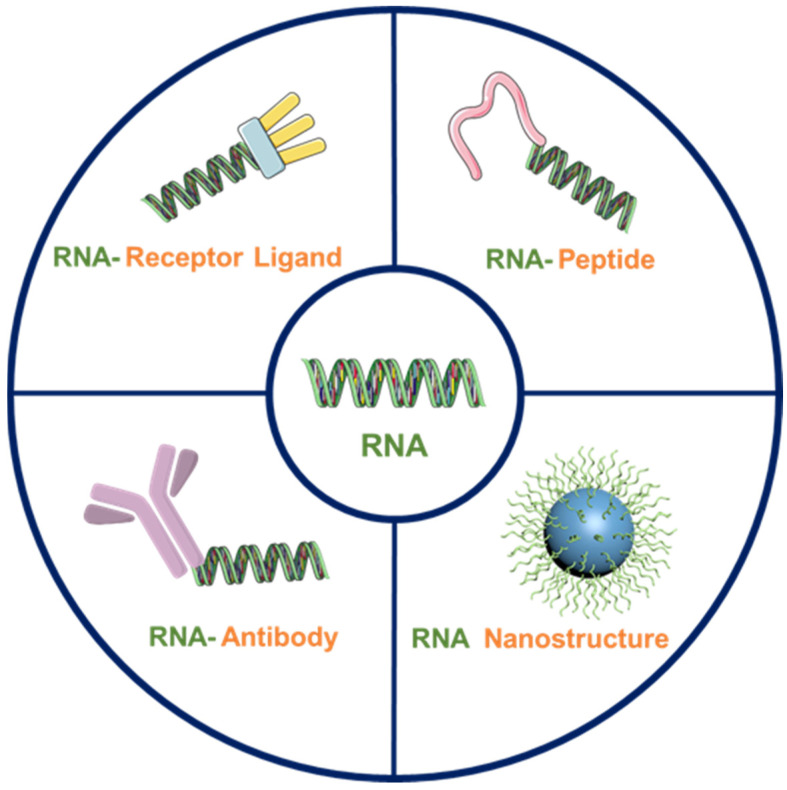
RNAs are modified with 4 types of junction molecules to form RNA-X conjugates.

Various biomolecules have been conjugated to noncoding RNAs such as siRNA to give RNA-X conjugates. Biomolecules including antibodies, membrane receptor ligands, targeting peptides and aptamers conjugated to siRNA were functional with respect to target recognition and membrane penetration (Figure 3) [72]. Due to endogenous transport mechanisms, some proteins and peptides are able to penetrate into cells and bring in a number of other molecules at the same time. Coupling targeted functional peptides with therapeutic RNA drugs can aid RNA to enter cells and perform biological functions [73]. For example, the cRGD (arginine-glycine-aspartate) cyclic peptide can bind tightly to the transmembrane glycoprotein αvβ3 integrin, which is highly expressed on a variety of tumor cells. Rudy and co-works covalently coupled bivalent, trivalent and tetravalent cRGD ligands to the 3’ end of the luciferase siRNA sense strand and demonstrated that high-affinity cRGD ligands strongly and specifically enhance siRNA uptake and inhibit intracellular luciferase expression in cells expressing αvβ3 integrin [74]. Skin penetration and cell entry (SPACE) peptides, with the ability to enhance the penetration of small molecules and protein cargos through the stratum corneum into the epidermis and dermis, also serve as excellent candidates for siRNA and miRNA delivery. Samir et al. coupled SPACE peptides with IL-10 siRNA and GAPDH siRNA, respectively, to downregulate IL-10 and GAPDH in animal epidermal cells [75]. By coupling with these peptides, the targeting and membrane penetrating abilities of RNA are significantly enhanced, effectively strengthening the biological functions of RNA drugs.

Coupling RNAs to receptor ligands can effectively increase their specific targeting ability as well. N-acetylgalactosamine (GalNAc) is a sugar derivative of galactose presented on damaged glycoproteins. It is able to bind specifically to the trimeric desialic acid glycoprotein receptor (ASGPR) (Kd = 2.5 nM), which is highly expressed on the surface of liver cells. The strategy of modifying GalNAc on siRNA for targeted delivery has been widely used [76,77,78]. Revusiran, the first GalNAc-siRNA coupling that enters clinical trials, primarily targets the thyrotropin transport protein mRNA to inhibit gene expression for the treatment of hereditary transthyretin amyloidosis (hATTR). Clinical trial results showed that Revusiran achieved 55–90% reduction in serum TTR levels with good therapeutic effects [79]. Besides, folic acid (FA) is a small molecule that binds tightly to folate receptors (FRs) which are highly expressed on the surface of many malignant tumors. Due to its small size, low immunogenicity, in vivo stability and strong binding affinity for FRs (Kd = 0.1–1 nM), FA has attracted a lot of attention for targeted siRNA delivery [80]. For instance, Thomas and his co-workers found that siRNAs modified with FA accumulated more efficiently in mouse tumors in a dose-dependent manner. However, due to the complexity of the synthesis of FA and siRNA, the application of folate-modified siRNA is currently only at the laboratory stage [81].

Although naked oligonucleotides are susceptible to degradation by nucleases, the stability can be greatly increased by forming spherical nucleic acids via nanotechnology, which allows the co-delivery of nucleic acid drugs with other types of drugs. Shi et al. constructed a siRNA vesicle-based drug delivery nanostructure, which was obtained by the self-assembly of siRNA-disulfide-poly (N-isopropylacrylamide) (siRNA–SS–PNIPAM) diblock copolymers. By exploiting temperature changes and intracellular reduction reactions, the loading of chemotherapeutic drugs into siRNA vesicles and the release of drugs from cells can be effectively controlled. Results from MCF-7 cancer cells and mouse tumor models showed that Dox-loaded siRNA nanovesicles could effectively inhibit tumor cell growth [82]. Zhang et al. reported a novel photo-unstable spherical nucleic acid (PSNA). The monomer was formed from hydrophilic HIF-1α siRNA and hydrophobic Bcl-2 peptide nucleic acid (pASO) through a single linear oxygen cleavable linker. After self-assembly based on different hydrophilicity and hydrophobicity, PSNAs were able to encapsulate photosensitizers (PS) to form nanoparticles. Under near-infrared light irradiation, the singlet oxygen produced by the photosensitizer was able to cleave the linker and release siRNA with pASO to target the gene. In Hela cell and mouse tumor models, HIF-1α mRNA was reduced by 79% and Bcl-2 mRNA was reduced by 67% in the light irradiated group of PSNA. This design combined nucleic acid therapy with photodynamic therapy and effectively enhance the efficacy of anti-tumor treatment [83].

Antibodies are biomolecules with high specificity, high stability and long in vivo half-life. Combination with antibodies can significantly improve the pharmacokinetics of drugs and reduce off-target rates. Antibody-drug conjugates (ADCs) have been rapidly developed in recent years. Antibody-siRNA conjugates (ARCs) have been successfully used to deliver siRNA to target cells expressing specific antigens, which is an effective strategy for RNA delivery [84]. Zhang’s team reported a novel photoresponsive antibody-siRNA conjugates (PARCs). It consisted of an anti-programmed death ligand 1 (αPD-L1) antibody, a photoreactive bond-breaking linker and a PD-L1 targeting siRNA (siPD-L1). Following systemic administration, PARC was able to bind specifically to PD-L1 on the surface of cancer cells, causing a revival of immune cell activity. After antibody-mediated endocytosis, light irradiation of the tumor was able to break the cleavable linker in PARC and release siPD-L1 to inhibit intracellular PD-L1 mRNA expression, preventing the continued production of PD-L1 [85]. Later, this team proceeded to report a ROS-responsive antibody-siRNA coupling, TCARROS. It consisted of an anti-programmed death receptor 1 (PD-1) antibody, a ROS-sensitive linker and siRNA targeting the CD38 gene. In contrast to PARCs, TCARROS primarily targets T cells and restores their immunological function. After systemic administration, TCARROS was able to bind to PD-1 on T cells and undergo bond breaking in the presence of large amounts of ROS in activated T cells, releasing functional siRNA and inhibiting CD38 mRNA expression. This therapy showed good synergistic therapeutic effects in mice transplanted with B16 melanoma and held potential for the development of combination of protein-targeted and nucleic acid therapy [86].

## 6. Small-Molecule Regulators of Noncoding RNAs

Small molecules that are capable of targeting or regulating RNAs are important intracellular molecular tools, for the understanding of underlying principles of specific RNA-involved interactions, also for demonstrating clinical efficacy [87,88]. For noncoding RNAs, especially small noncoding RNA such as miRNA, varieties of small molecules have been found to regulate the expression or activities of the disease-related miRNAs [64,89,90]. Reporter systems with readily read-out signals such as luciferase expression have been engineered in live cells for the screening of small molecules with regulatory effects on various miRNAs [91]. In these screening models, small molecules from different sources were incubated with cells transfected with the reporter systems. The effect of specific molecule on the expression or activity of the miRNA with sequence complementary to the epitope engineered at the 3’-UTR of luciferase reporter gene was then evaluated on live cells. Active molecules that showed specific regulatory activity on one type of miRNAs, such as myogenic miRNAs, were used as small-molecule probe to elucidate unknown miRNA-involved regulatory network in live cells [92,93].

Small molecule compounds targeting oncogenic miRNAs were considered to hold potentials for cancer therapy [94]. Using lentiviral reporter constructs in which the complementary sequences to those of oncogenic miR-21 down-stream of a luciferase reporter gene, Deiters et.al. screened compounds from a library containing more than 1000 compounds and obtained one specific and efficient inhibitor of miR-21 expression [95]. They then revealed that small molecule inhibition of miR-21 expression reduced cell viability and microtumor formation [96], or rescued chemosensitivity of renal-cell carcinoma to topotecan [97]. The biogenesis or maturation of miRNAs were found to be able to regulated by molecules such as Enoxacin [98], Tetracyclines [99] or cyclic peptidomimetics [100]. Small-molecule inhibitor that modulates miRNA biogenesis was reported to disrupt TRBP-Dicer interaction against hepatocellular carcinoma [101]. Bifunctional small molecules were also designed to regulate miR-21 biogenesis [102], in which a pre-miRNA binding unit was connected by a linker to a Dicer inhibiting unit. Discovery of more small molecules with regulatory effect on miRNAs might be aided by newly-developed methods, such as mirror image phage display [103]. Methods to predict the potentials of small molecules to associate miRNAs have also been developed using graphlet interaction [104].

## 7. Ribonuclease Targeting Chimeras (RIBOTAC) as Emerging Molecules to Degrade Noncoding RNAs

**Figure 4:**
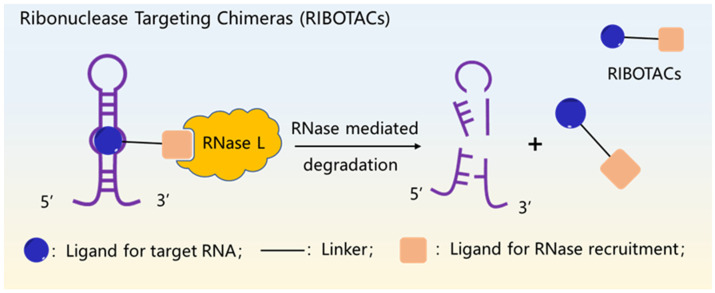
The mechanism of RIBOTACs.

Among various small molecule targeting strategies, RIBOTAC is entering the limelight as a novel intracellular regulatory tool. Similar to proteolysis-targeting chimera (PROTAC), RIBOTAC is a bifunctional small molecule, with one part specifically targeting RNAs secondary or tertiary structures, and the other part recruiting and activating RNaseL enzymes to induce degradation of noncoding RNAs (Figure 4). By using different RNA ligands, various RIBOTACs small molecules can be used to degrade different noncoding RNAs, enabling precise regulation of RNAs at the small molecule level [105,106].

Studies have shown that approximately 50% of the nucleotides in RNA targets are structured or non-classically paired, and therefore approximately half of the sequences cannot be targeted by sequence recognition methods [107]. In addition, many structured regions have been demonstrated to regulate the biological function of RNA, and studies have shown that many sites are associated with the development of diseases [108]. RIBOTAC is a novel therapeutic technology that targets structured regions of RNA, effectively solving the problem that traditional nucleic acid drugs failed to target non-classical paired regions and providing a new strategy for the design of RNA-targeted drugs [109].

Dr. Matthew D. Disney and his team first developed a strategy to design structure-specific ligands based on RNA sequences, and named it Inforna. Inforna integrated a selection-based strategy (two-dimensional combinatorial screening), a statistical approach, and the structural information about RNA targets of interest to allow computational and high-throughput screening of small molecules with the strongest affinity for structural sites. Compared to traditional methods, Inforna-based screening is more systematic and reliable, and is now being used for the development of new targets in RNA structural regions [110].

Based on the Inforna platform, Disney developed the earliest RIBOTACs small molecules in 2018. The initial RIBOTACs consisted of two parts, one of which was Targaprimir-96 (TGP-96) small molecule that specifically targets the primary transcript of miR-96 (pri-miR-96), and the other was a short 2′-5′ poly(A) oligonucleotide (2′-5′ A4) that recruits RNaseL enzyme. The results showed that after targeted degradation of Pri-miR-96 by RIBOTACs, downstream miR-96 expression was downregulated, which in turn inhibited the expression of the pro-apoptotic FOXO1 transcription factor and induced apoptosis in breast cancer cells [111]. In 2019, another RIBOTAC molecule specifically targeting and degrading Pre-microRNA-210 (pre-miR-210) was designed. The molecule consists of a Targaprimir-210 (TGP-210) molecule targeting the pre-miR-210 at the Dicer enzyme processing site with a 2′-5′A4. Using small molecule-targeted inhibition of TGP-210 alone or molecularly targeted degradation of RIBOTAC, they blocked the synthesis of downstream mature miR-210-3p [112]. In 2020, a new set of RIBOTAC molecules for SARS-CoV-2 viral RNA was developed. The frameshifting element (FSE) in the SARS-CoV-2 RNA fragment controls the translation of pp1a and pp1ab polyproteins, both of which are essential for viral replication. Enhancing the thermodynamic stability of the FSE region can reduce the efficiency of frameshifting and inhibit rapid viral translation. Disney’s team used Inforna to screen for small molecules C5 that specifically target the FSE attenuator hairpin structure, and covalently bind to small molecules that recruit the RNaseL enzyme to form the RIBOTAC molecule C5-RIBOTAC. The results of the study indicated that C5-RIBOTAC could directly recruit RNaseL enzyme to induce the degradation of SARS-CoV-2 viral RNA fragments, paving a new path for development of new coronavirus therapeutic agents [113].

Compared with traditional nucleic acid drugs, RIBOTAC is a small molecule with smaller molecular weight, higher stability and better membrane penetration effects. At the same time, RIBOTAC uses structural regions as its own targeting sites, which greatly broadens the RNA drug-forming targets and provides more possibilities for the development of novel RNA-targeted drugs. RIBOTAC molecules are capable of degrading the target RNA by recruiting RNaseL enzymes after targeting. Therefore, RIBOTAC molecules theoretically only require a catalytic number of drugs to initiate target degradation with less chance of acquiring drug resistance and better safety [107,108]. Despite these distinguishing advantages, RIBOTAC requires more complex design compared with traditional nucleic acid drugs, where only the functional sequences of target RNA needed to be considered. The screening of small molecules for targeting in RIBOTAC technology is difficult, and so far only a few small molecules with targeting functions have been screened by Disney’s team using the Inforna platform system, posing a serious challenge for the development of RIBOTAC [105].

## 8. Perspectives

Noncoding RNA-based nucleic acid drugs as next-generation drugs serve as an alternative approach for therapy. In particular, RNA-based drugs can theoretically target any gene of interest, which is “undruggable” for most small molecules and antibodies. Another feature is that the development of new nucleic acid drugs is simple and fast. It is straightforward to design new drugs towards target sequences based on Watson-Crick base pairing. In addition, nucleic acid-based therapies are more precise and efficient due to the sequence-target mechanism, but less susceptible to drug resistance [114,115].

Despite the outstanding properties, nucleic drugs also have some challenges. For example, the inherent instability of nucleic acid drugs makes it sensitive to either nucleic acid endonucleases or exonucleases and thus they are highly susceptible to degradation. In addition, how to effectively deliver oligonucleotides to target sites is another challenge. The instability, negative charge and hydrophilic nature of oligonucleotide hinder the diffusion through cell membranes [56,57,58]. In addition to chemical modification and RNA-X conjugates, using appropriate vehicles is another common strategy to increase the stability and cell-penetrating ability of RNA, and many delivery systems including viral vectors and non-viral vectors have been developed [116,117,118].

Lipid nanoparticles (LNP) are one of the most established vectors for nucleic acid drug delivery and have gained much attention as a delivery platform for COVID-19 mRNA vaccines [119,120]. In addition, exosomes are extracellular vesicles actively secreted by human cells and are capable of delivering various types of molecular signals to recipient cells and regulating their functions. With their natural affinity for recipient cells and their inherent ability to deliver nucleic acids, lipids and proteins between cells, exosomes serve as a class of promising RNA delivery vehicles. Zhang et al. facilitated in vivo delivery of siRNAs through circulating exosomes by reprogramming the host liver using gene circuits to direct the synthesis and self-assembly of siRNAs into secretory exosomes. This strategy took full advantage of the inherent characteristics of exosomes to protect and precisely deliver RNAs. Meanwhile, the in vivo synthesis of exosomes also circumvented the difficulties of large-scaled in vitro extraction of exosomes [121,122,123]. Moreover, cationic cell-penetrating peptides have become another efficient carrier for RNA drug delivery, with the advantages of high biocompatibility, good membrane penetration and easy synthesis. Nonaarginine (R9) is a common positively charged cell-penetrating peptide (CPP), which is able to self-assemble with negatively charged RNA by electrostatic force to form stable nanoparticles, guided by the targeting moiety to bring therapeutic RNA drugs into target cells. Zhang et al. modified R9 with targeting moiety FA and a hydrophobic peptide nucleic acid (PNA anti-miR-21). This vector can form nanoparticles with miR-34a and assist miR-34a and PNA anit-miR-21 to enter the Hela cell for dual gene therapy [124,125,126,127]. Hydrogels formed by the self-assembly of small molecules such as peptides have shown potentials in delivering the noncoding RNAs mixed in the hydrogel matrix into live cells. Cationic polymer- and lipid-independent siRNA nanogel and intercalation-driven formation of siRNA nanogels with good stability have been reported to deliver siRNAs into cells through different endocytic pathways to silence target genes [128,129,130,131,132].

With the continuous development of RNA modification and regulation technology, noncoding RNA-based nucleic acid drugs will have a wide scope for development. A variety of diseases caused by non-druggable proteins will have new treatments, and we expect more nucleic acid drugs to be approved and marketed in the future to bring benefits to patients.

## Figures and Tables

**Table 1:** Nucleic acid drugs approved by FDA and EMA.

Type	Drug Name	Approval	Indication	Company
Antisense Oligonucleotide	Vitravene (Fomivirsen)	1998 (Withdrawn)	Cytomegalovirus retinitis	Isis Pharmaecuticals/Novartis Ophthalmics
	Kynamro (Mipomersen)	2013	Homozygous familial hypercholesterolemia	Kastle Therapeutics
	Exondys 51 (Eteplirsen)	2016	Duchenne muscular dystrophy	Sarepta Therapeutics
	Spinraza (Nusinersen)	2016	Spinal muscular atrophy	Biogen
	Tegsedi (Inotersen)	2018	Familial amyloid neuropathies	Akcea Therapeutics
	Vyondys 53 (Golodirsen)	2019	Duchenne muscular dystrophy	Sarepta Therapeutics
	Waylivra (Volanesorsen)	2019	Familial chylomicronemia syndrome	Akcea Therapeutics
	Viltepso (Viltolarsen)	2020	Duchenne muscular dystrophy	Nippon Shinyaku with NCNP
	Amondys 45 (Casimersen)	2021	Duchenne muscular dystrophy	Sarepta Therapeutics
siRNA	Onpattro (Patisiran)	2018	Familial amyloid neuropathies	Alnylam Pharmaceuticals
	Givlaari (Givosiran)	2019	Acute hepatic porphyria	Alnylam Pharmaceuticals
	Oxlumo (Lumasiran)	2020	Primary hyperoxaluria type 1	Alnylam Pharmaceuticals
	Leqvio (Inclisiran)	2021	Lower LDL cholesterol	Novartis
	Amvuttra (Vutrisiran)	2022	Hereditary transthyretin-mediated amyloid polyneuropathy	Alnylam Pharmaceuticals
Oligonucleotide	Macugen (Pegaptanib)	2004	Age-related macular degeneration (AMD) of the retina	OSI Pharmaceuticals
	Defitelio (Defibrotide sodium)	2016	Hepatic veno-occlusive disease	Jazz Pharmaceuticals Plc

## Data Availability

Not applicable.

## References

[1] Comfort N. (2015). Genetics: We are the 98%. Nature.

[2] Anastasiadou E., Jacob L.S., Slack F.J. (2018). Non-coding RNA networks in cancer. Nat. Rev. Cancer.

[3] Hombach S., Kretz M. (2016). Non-coding RNAs: Classification, Biology and Functioning. Non-Coding RNAs in Colorectal Cancer.

[4] Detomas M., Pivonello C., Pellegrini B., Landwehr L.S., Sbiera S., Pivonello R., Ronchi C.L., Colao A., Altieri B., De Martino M.C. (2022). MicroRNAs and Long Non-Coding RNAs in Adrenocortical Carcinoma. Cells.

[5] Liu S.J., Dang H.X., Lim D.A., Feng F.Y., Maher C.A. (2021). Long noncoding RNAs in cancer metastasis. Nat. Rev. Cancer.

[6] Riquelme I., Perez-Moreno P., Letelier P., Brebi P., Roa J.C. (2021). The Emerging Role of PIWI-Interacting RNAs (piRNAs) in Gastrointestinal Cancers: An Updated Perspective. Cancers.

[7] Szczepanek J., Skorupa M., Tretyn A. (2022). MicroRNA as a Potential Therapeutic Molecule in Cancer. Cells.

[8] Kaikkonen M.U., Adelman K. (2018). Emerging Roles of Non-Coding RNA Transcription. Trends Biochem. Sci..

[9] Wu P., Mo Y., Peng M., Tang T., Zhong Y., Deng X., Xiong F., Guo C., Wu X., Li Y. (2020). Emerging role of tumor-related functional peptides encoded by lncRNA and circRNA. Mol. Cancer.

[10] Raj D.K., Das Mohapatra A., Jnawali A., Zuromski J., Jha A., Cham-Kpu G., Sherman B., Rudlaff R.M., Nixon C.E., Hilton N. (2020). Anti-PfGARP activates programmed cell death of parasites and reduces severe malaria. Nature.

[11] Mehta A., Baltimore D. (2016). MicroRNAs as regulatory elements in immune system logic. Nat. Rev. Immunol..

[12] Tuorto F., Parlato R. (2019). rRNA and tRNA Bridges to Neuronal Homeostasis in Health and Disease. J. Mol. Biol..

[13] Giustacchini A., Nucera S., Lechman E.R., Fanciullo C., Boccalatte F.E., Plati T., Ranghetti A., Vago L., Bernardi M., Ponzoni M. (2013). A Mechanistic Role For Mir-126, a Hematopoietic Stem Cell MicroRNA, In Acute Leukemias. Blood.

[14] He L., Thomson J.M., Hemann M.T., Hernando-Monge E., Mu D., Goodson S., Powers S., Cordon-Cardo C., Lowe S.W., Hannon G.J. (2005). A microRNA polycistron as a potential human oncogene. Nature.

[15] Ren C.X., Leng R.X., Fan Y.G., Pan H.F., Wu C.H., Ye D.Q. (2016). MicroRNA-210 and its theranostic potential. Expert Opin. Ther. Targets.

[16] Arghiani N., Matin M.M. (2021). miR-21: A Key Small Molecule with Great Effects in Combination Cancer Therapy. Nucleic Acid Ther..

[17] Luan S.H., Sun L.L., Huang F.P. (2010). MicroRNA-34a: A Novel Tumor Suppressor in p53-mutant Glioma Cell Line U251. Arch. Med. Res..

[18] Chirshev E., Oberg K.C., Ioffe Y.J., Unternaehrer J.J. (2019). Let-7 as biomarker, prognostic indicator, and therapy for precision medicine in cancer. Clin. Transl. Med..

[19] Toiyama Y., Hur K., Tanaka K., Inoue Y., Kusunoki M., Boland C.R., Goel A. (2014). Serum miR-200c Is a Novel Prognostic and Metastasis-Predictive Biomarker in Patients With Colorectal Cancer. Ann. Surg..

[20] Bandiera S., Pfeffer S., Baumert T.F., Zeisel M.B. (2015). miR-122-A key factor and therapeutic target in liver disease. J. Hepatol..

[21] Galvao-Lima L.J., Morais A.H.F., Valentim R.A.M., Barreto E.J.S.S. (2021). miRNAs as biomarkers for early cancer detection and their application in the development of new diagnostic tools. Biomed. Eng. Online.

[22] Moatar A.I., Chis A.R., Marian C., Sirbu I.O. (2022). Gene Network Analysis of the Transcriptome Impact of SARS-CoV-2 Interacting MicroRNAs in COVID-19 Disease. Int. J. Mol. Sci..

[23] Huang W. (2017). MicroRNAs: Biomarkers, Diagnostics, and Therapeutics. Methods Mol. Biol..

[24] Garnier N., Pollet K., Fourcot M., Caplan M., Marot G., Goutay J., Labreuche J., Soncin F., Boukherroub R., Hober D. (2022). Altered microRNA expression in severe COVID-19: Potential prognostic and pathophysiological role. Clin. Transl. Med..

[25] Giannella A., Riccetti S., Sinigaglia A., Piubelli C., Razzaboni E., Di Battista P., Agostini M., Dal Molin E., Manganelli R., Gobbi F. (2022). Circulating microRNA signatures associated with disease severity and outcome in COVID-19 patients. Front. Immunol..

[26] Baptista B., Riscado M., Queiroz J.A., Pichon C., Sousa F. (2021). Non-coding RNAs: Emerging from the discovery to therapeutic applications. Biochem. Pharmacol..

[27] Yang H., Qi C., Li B., Cheng L. (2022). Non-coding RNAs as Novel Biomarkers in Cancer Drug Resistance. Curr. Med. Chem..

[28] Matsui M., Corey D.R. (2017). Non-coding RNAs as drug targets. Nat. Rev. Drug Discov..

[29] Aguilar R., Spencer K.B., Kesner B., Rizvi N.F., Badmalia M.D., Mrozowich T., Mortison J.D., Rivera C., Smith G.F., Burchard J. (2022). Targeting Xist with compounds that disrupt RNA structure and X inactivation. Nature.

[30] Kulkarni J.A., Witzigmann D., Thomson S.B., Chen S., Leavitt B.R., Cullis P.R., van der Meel R. (2021). The current landscape of nucleic acid therapeutics. Nat. Nanotechnol..

[31] Friedmann T., Roblin R. (1972). Gene therapy for human genetic disease?. Science.

[32] Castanotto D., Rossi J.J. (2009). The promises and pitfalls of RNA-interference-based therapeutics. Nature.

[33] Wilson R.C., Doudna J.A. (2013). Molecular mechanisms of RNA interference. Annu. Rev. Biophys..

[34] Adams D., Gonzalez-Duarte A., O’Riordan W.D., Yang C.C., Ueda M., Kristen A.V., Tournev I., Schmidt H.H., Coelho T., Berk J.L. (2018). Patisiran, an RNAi Therapeutic, for Hereditary Transthyretin Amyloidosis. N. Engl. J. Med..

[35] Bartel D.P. (2004). MicroRNAs: Genomics, biogenesis, mechanism, and function. Cell.

[36] Zhu Y., Zhu L., Wang X., Jin H. (2022). RNA-based therapeutics: An overview and prospectus. Cell Death Dis..

[37] Ghanbarian H., Aghamiri S., Eftekhary M., Wagner N., Wagner K.D. (2021). Small Activating RNAs: Towards the Development of New Therapeutic Agents and Clinical Treatments. Cells.

[38] Tan C.P., Sinigaglia L., Gomez V., Nicholls J., Habib N.A. (2021). RNA Activation-A Novel Approach to Therapeutically Upregulate Gene Transcription. Molecules.

[39] Sarker D., Plummer R., Meyer T., Sodergren M.H., Basu B., Chee C.E., Huang K.W., Palmer D.H., Ma Y.T., Evans T.R.J. (2020). MTL-CEBPA, a Small Activating RNA Therapeutic Upregulating C/EBP-alpha, in Patients with Advanced Liver Cancer: A First-in-Human, Multicenter, Open-Label, Phase I Trial. Clin. Cancer Res..

[40] Bennett C.F., Baker B.F., Pham N., Swayze E., Geary R.S. (2017). Pharmacology of Antisense Drugs. Annu. Rev. Pharmacol. Toxicol..

[41] Rossor A.M., Reilly M.M., Sleigh J.N. (2018). Antisense oligonucleotides and other genetic therapies made simple. Pract. Neurol..

[42] Dhuri K., Bechtold C., Quijano E., Pham H., Gupta A., Vikram A., Bahal R. (2020). Antisense Oligonucleotides: An Emerging Area in Drug Discovery and Development. J. Clin. Med..

[43] Gragoudas E.S., Adamis A.P., Cunningham E.T., Feinsod M., Guyer D.R. (2004). Pegaptanib for Neovascular Age-Related Macular Degeneration. N. Engl. J. Med..

[44] Ng E.W., Shima D.T., Calias P., Cunningham E.T., Guyer D.R., Adamis A.P. (2006). Pegaptanib, a targeted anti-VEGF aptamer for ocular vascular disease. Nat. Rev. Drug Discov..

[45] Ning L., Liu M., Gou Y., Yang Y., He B., Huang J. (2022). Development and application of ribonucleic acid therapy strategies against COVID-19. Int. J. Biol. Sci..

[46] Rossi J.J. (1998). Therapeutic Ribozymes: Principles and Applications. BioDrugs.

[47] Garbo S., Maione R., Tripodi M., Battistelli C. (2022). Next RNA Therapeutics: The Mine of Non-Coding. Int. J. Mol. Sci..

[48] Feng R., Patil S., Zhao X., Miao Z., Qian A. (2021). RNA Therapeutics—Research and Clinical Advancements. Front. Mol. Biosci..

[49] Aquino-Jarquin G. (2020). Novel Engineered Programmable Systems for ADAR-Mediated RNA Editing. Mol. Ther. Nucleic Acids.

[50] Chaput J.C., Herdewijn P. (2019). What Is XNA?. Angew. Chem. Int. Ed. Engl..

[51] Wang Y., Wang Y., Song D., Sun X., Zhang Z., Li X., Li Z., Yu H. (2021). A Threose Nucleic Acid Enzyme with RNA Ligase Activity. J. Am. Chem. Soc..

[52] Wang Y., Wang Y., Song D., Sun X., Li Z., Chen J.Y., Yu H. (2022). An RNA-cleaving threose nucleic acid enzyme capable of single point mutation discrimination. Nat. Chem..

[53] Dolgin E. (2022). tRNA therapeutics burst onto startup scene. Nat. Biotechnol..

[54] Damase T.R., Sukhovershin R., Boada C., Taraballi F., Pettigrew R.I., Cooke J.P. (2021). The Limitless Future of RNA Therapeutics. Front. Bioeng. Biotechnol..

[55] Kotowska-Zimmer A., Pewinska M., Olejniczak M. (2021). Artificial miRNAs as therapeutic tools: Challenges and opportunities. Wiley Interdiscip. Rev. RNA.

[56] Hu B., Zhong L., Weng Y., Peng L., Huang Y., Zhao Y., Liang X.J. (2020). Therapeutic siRNA: State of the art. Signal. Transduct. Target. Ther..

[57] Neumeier J., Meister G. (2020). siRNA Specificity: RNAi Mechanisms and Strategies to Reduce Off-Target Effects. Front. Plant. Sci..

[58] Gupta A., Andresen J.L., Manan R.S., Langer R. (2021). Nucleic acid delivery for therapeutic applications. Adv. Drug Deliv. Rev..

[59] Winkle M., El-Daly S.M., Fabbri M., Calin G.A. (2021). Noncoding RNA therapeutics—Challenges and potential solutions. Nat. Rev. Drug Discov..

[60] Chen Y., Hong T., Wang S., Mo J., Tian T., Zhou X. (2017). Epigenetic modification of nucleic acids: From basic studies to medical applications. Chem. Soc. Rev..

[61] Shen X., Corey D.R. (2018). Chemistry, mechanism and clinical status of antisense oligonucleotides and duplex RNAs. Nucleic Acids Res..

[62] Flamme M., McKenzie L.K., Sarac I., Hollenstein M. (2019). Chemical methods for the modification of RNA. Methods.

[63] Rozners E. (2022). Chemical Modifications of CRISPR RNAs to Improve Gene-Editing Activity and Specificity. J. Am. Chem. Soc..

[64] Li J., Tan S., Kooger R., Zhang C., Zhang Y. (2014). MicroRNAs as novel biological targets for detection and regulation. Chem. Soc. Rev..

[65] Hagedorn P.H., Persson R., Funder E.D., Albaek N., Diemer S.L., Hansen D.J., Moller M.R., Papargyri N., Christiansen H., Hansen B.R. (2018). Locked nucleic acid: Modality, diversity, and drug discovery. Drug Discov. Today.

[66] Pasternak A., Wengel J. (2011). Unlocked nucleic acid—An RNA modification with broad potential. Org. Biomol. Chem..

[67] Zhang J., Zheng J., Lu C., Du Q., Liang Z., Xi Z. (2012). Modification of the siRNA passenger strand by 5-nitroindole dramatically reduces its off-target effects. ChemBioChem.

[68] Wahba A.S., Azizi F., Deleavey G.F., Brown C., Robert F., Carrier M., Kalota A., Gewirtz A.M., Pelletier J., Hudson R.H. (2011). Phenylpyrrolocytosine as an unobtrusive base modification for monitoring activity and cellular trafficking of siRNA. ACS Chem. Biol..

[69] Smith C.I.E., Zain R. (2019). Therapeutic Oligonucleotides: State of the Art. Annu. Rev. Pharmacol. Toxicol..

[70] Shadid M., Badawi M., Abulrob A. (2021). Antisense oligonucleotides: Absorption, distribution, metabolism, and excretion. Expert Opin. Drug Metabol. Toxicol..

[71] Selvam C., Mutisya D., Prakash S., Ranganna K., Thilagavathi R. (2017). Therapeutic potential of chemically modified siRNA: Recent trends. Chem. Biol. Drug Des..

[72] Chernikov I.V., Vlassov V.V., Chernolovskaya E.L. (2019). Current Development of siRNA Bioconjugates: From Research to the Clinic. Front. Pharmacol..

[73] Klabenkova K., Fokina A., Stetsenko D. (2021). Chemistry of Peptide-Oligonucleotide Conjugates: A Review. Molecules.

[74] Alam M.R., Ming X., Fisher M., Lackey J.G., Rajeev K.G., Manoharan M., Juliano R.L. (2011). Multivalent cyclic RGD conjugates for targeted delivery of small interfering RNA. Bioconjug. Chem..

[75] Hsu T., Mitragotri S. (2011). Delivery of siRNA and other macromolecules into skin and cells using a peptide enhancer. Proc. Natl. Acad. Sci. USA.

[76] Springer A.D., Dowdy S.F. (2018). GalNAc-siRNA Conjugates: Leading the Way for Delivery of RNAi Therapeutics. Nucleic Acid Ther..

[77] Thangamani L., Balasubramanian B., Easwaran M., Natarajan J., Pushparaj K., Meyyazhagan A., Piramanayagam S. (2021). GalNAc-siRNA conjugates: Prospective tools on the frontier of anti-viral therapeutics. Pharmacol. Res..

[78] Yan Y., Liu X.Y., Lu A., Wang X.Y., Jiang L.X., Wang J.C. (2022). Non-viral vectors for RNA delivery. J. Control. Release.

[79] Zimmermann T.S., Karsten V., Chan A., Chiesa J., Boyce M., Bettencourt B.R., Hutabarat R., Nochur S., Vaishnaw A., Gollob J. (2017). Clinical Proof of Concept for a Novel Hepatocyte-Targeting GalNAc-siRNA Conjugate. Mol. Ther..

[80] Gangopadhyay S., Nikam R.R., Gore K.R. (2021). Folate Receptor-Mediated siRNA Delivery: Recent Developments and Future Directions for RNAi Therapeutics. Nucleic Acid Ther..

[81] Thomas M., Kularatne S.A., Qi L., Kleindl P., Leamon C.P., Hansen M.J., Low P.S. (2009). Ligand-targeted delivery of small interfering RNAs to malignant cells and tissues. Ann. N. Y. Acad. Sci..

[82] Zheng M., Jiang T., Yang W., Zou Y., Wu H., Liu X., Zhu F., Qian R., Ling D., McDonald K. (2019). The siRNAsome: A Cation-Free and Versatile Nanostructure for siRNA and Drug Co-delivery. Angew. Chem. Int. Ed. Engl..

[83] Chen L., Li G., Wang X., Li J., Zhang Y. (2021). Spherical Nucleic Acids for Near-Infrared Light-Responsive Self-Delivery of Small-Interfering RNA and Antisense Oligonucleotide. ACS Nano.

[84] Dugal-Tessier J., Thirumalairajan S., Jain N. (2021). Antibody-Oligonucleotide Conjugates: A Twist to Antibody-Drug Conjugates. J. Clin. Med..

[85] Wang X., Xiao X., Feng Y., Li J., Zhang Y. (2022). A photoresponsive antibody-siRNA conjugate for activatable immunogene therapy of cancer. Chem. Sci..

[86] Wang X., Wang Y., Li J., Tian T., Li J., Guo Z., Zhang Y. (2021). T Cell-Signaling-Responsive Conjugate of Antibody with siRNA to Overcome Acquired Resistance to anti-PD-1 Immunotherapy. Adv. Ther..

[87] Falese J.P., Donlic A., Hargrove A.E. (2021). Targeting RNA with small molecules: From fundamental principles towards the clinic. Chem. Soc. Rev..

[88] Li J., Kong H., Huang L., Cheng B., Qin K., Zheng M., Yan Z., Zhang Y. (2018). Visible Light-Initiated Bioorthogonal Photoclick Cycloaddition. J. Am. Chem. Soc..

[89] Van Meter E.N., Onyango J.A., Teske K.A. (2020). A review of currently identified small molecule modulators of microRNA function. Eur. J. Med. Chem.

[90] Li J., Zhang W., Zhou M., Kooger R., Zhang Y. (2013). Small Molecules Modulating Biogenesis or Processing of microRNAs with Therapeutic Potentials. Curr. Med. Chem..

[91] Chen X.J., Huang C.M., Zhang W.J., Wu Y.H., Chen X., Zhang C.Y., Zhang Y. (2012). A universal activator of microRNAs identified from photoreaction products. Chem. Commun..

[92] Tan S.B., Huang C., Chen X., Wu Y., Zhou M., Zhang C., Zhang Y. (2013). Small molecular inhibitors of miR-1 identified from photocycloadducts of acetylenes with 2-methoxy-1,4-naphthalenequinone. Bioorg. Med. Chem..

[93] Tan S.B., Li J., Chen X., Zhang W., Zhang D., Zhang C., Li D., Zhang Y. (2014). Small molecule inhibitor of myogenic microRNAs leads to a discovery of miR-221/222-myoD-myomiRs regulatory pathway. Chem. Biol..

[94] Monroig Pdel C., Chen L., Zhang S., Calin G.A. (2015). Small molecule compounds targeting miRNAs for cancer therapy. Adv. Drug Deliv. Rev..

[95] Gumireddy K., Young D.D., Xiong X., Hogenesch J.B., Huang Q., Deiters A. (2008). Small-molecule inhibitors of microRNA miR-21 function. Angew. Chem. Int. Ed. Engl..

[96] Ankenbruck N., Kumbhare R., Naro Y., Thomas M., Gardner L., Emanuelson C., Deiters A. (2019). Small molecule inhibition of microRNA-21 expression reduces cell viability and microtumor formation. Bioorg. Med. Chem..

[97] Naro Y., Ankenbruck N., Thomas M., Tivon Y., Connelly C.M., Gardner L., Deiters A. (2018). Small Molecule Inhibition of MicroRNA miR-21 Rescues Chemosensitivity of Renal-Cell Carcinoma to Topotecan. J. Med. Chem..

[98] Felicetti T., Cecchetti V., Manfroni G. (2020). Modulating microRNA Processing: Enoxacin, the Progenitor of a New Class of Drugs. J. Med. Chem..

[99] Garner A.L., Lorenz D.A., Sandoval J., Gallagher E.E., Kerk S.A., Kaur T., Menon A. (2019). Tetracyclines as Inhibitors of Pre-microRNA Maturation: A Disconnection between RNA Binding and Inhibition. ACS Med. Chem. Lett..

[100] Yan H., Zhou M., Bhattarai U., Song Y.B., Zheng M.M., Cai J.F., Liang F.S. (2019). Cyclic Peptidomimetics as Inhibitor for miR-155 Biogenesis. Mol. Pharm..

[101] Peng T., He Y.J., Wang T., Yu J.L., Ma X.F., Zhou Z.Y., Sheng Y.W., Li L.Y., Peng H.P., Li S. (2022). Discovery of a Novel Small-Molecule Inhibitor Disrupting TRBP- Dicer Interaction against Hepatocellular Carcinoma via the Modulation of microRNA Biogenesis. J. Med. Chem..

[102] Yan H., Bhattarai U., Guo Z.F., Liang F.S. (2017). Regulating miRNA-21 Biogenesis By Bifunctional Small Molecules. J. Am. Chem. Soc..

[103] Sakamoto K., Otake K., Umemoto T. (2017). Discovery of peptidic miR-21 processing inhibitor by mirror image phage display: A novel method to generate RNA binding D-peptides. Bioorg. Med. Chem. Lett..

[104] Guan N.N., Sun Y.Z., Ming Z., Li J.Q., Chen X. (2018). Prediction of Potential Small Molecule-Associated MicroRNAs Using Graphlet Interaction. Front. Pharmacol..

[105] Costales M.G., Childs-Disney J.L., Haniff H.S., Disney M.D. (2020). How We Think about Targeting RNA with Small Molecules. J. Med. Chem..

[106] Dey S.K., Jaffrey S.R. (2019). RIBOTACs: Small Molecules Target RNA for Degradation. Cell Chem. Biol..

[107] Stombaugh J., Zirbel C.L., Westhof E., Leontis N.B. (2009). Frequency and isostericity of RNA base pairs. Nucleic Acids Res..

[108] Crews L.A., Balaian L., Delos Santos N.P., Leu H.S., Court A.C., Lazzari E., Sadarangani A., Zipeto M.A., La Clair J.J., Villa R. (2016). RNA Splicing Modulation Selectively Impairs Leukemia Stem Cell Maintenance in Secondary Human AML. Cell Stem Cell.

[109] Liu X., Haniff H.S., Childs-Disney J.L., Shuster A., Aikawa H., Adibekian A., Disney M.D. (2020). Targeted Degradation of the Oncogenic MicroRNA 17-92 Cluster by Structure-Targeting Ligands. J. Am. Chem. Soc..

[110] Disney M.D., Winkelsas A.M., Velagapudi S.P., Southern M., Fallahi M., Childs-Disney J.L. (2016). Inforna 2.0: A Platform for the Sequence-Based Design of Small Molecules Targeting Structured RNAs. ACS Chem. Biol..

[111] Costales M.G., Matsumoto Y., Velagapudi S.P., Disney M.D. (2018). Small Molecule Targeted Recruitment of a Nuclease to RNA. J. Am. Chem. Soc..

[112] Costales M.G., Suresh B., Vishnu K., Disney M.D. (2019). Targeted Degradation of a Hypoxia-Associated Non-coding RNA Enhances the Selectivity of a Small Molecule Interacting with RNA. Cell Chem. Biol..

[113] Haniff H.S., Tong Y., Liu X., Chen J.L., Suresh B.M., Andrews R.J., Peterson J.M., O’Leary C.A., Benhamou R.I., Moss W.N. (2020). Targeting the SARS-CoV-2 RNA Genome with Small Molecule Binders and Ribonuclease Targeting Chimera (RIBOTAC) Degraders. ACS Cent. Sci..

[114] Carthew R.W., Sontheimer E.J. (2009). Origins and Mechanisms of miRNAs and siRNAs. Cell.

[115] Hamid U.Z., Sim M.S., Guad R.M., Subramaniyan V., Sekar M., Fuloria N.K., Fuloria S., Choy K.W., Fareez I.M., Bonam S.R. (2022). Molecular Regulatory Roles of Long Non-coding RNA HOTTIP: An Overview in Gastrointestinal Cancers. Curr. Mol. Med..

[116] Shen W., Wang R., Fan Q., Gao X., Wang H., Shen Y., Li Y., Cheng Y. (2020). Natural Polyphenol Inspired Polycatechols for Efficient siRNA Delivery. CCS Chem..

[117] Ding P., Huang J., Wei C., Liu W., Zhou W., Wang J., Wang M., Guo X., Cohen Stuart M.A., Wang J. (2020). Efficient and Generic Preparation of Diverse Polyelectrolyte Nanogels by Electrostatic Assembly Directed Polymerization. CCS Chem..

[118] Eygeris Y., Gupta M., Kim J., Sahay G. (2022). Chemistry of Lipid Nanoparticles for RNA Delivery. Acc. Chem. Res..

[119] Vlatkovic I. (2021). Non-Immunotherapy Application of LNP-mRNA: Maximizing Efficacy and Safety. Biomedicines.

[120] Schoenmaker L., Witzigmann D., Kulkarni J.A., Verbeke R., Kersten G., Jiskoot W., Crommelin D.J.A. (2021). mRNA-lipid nanoparticle COVID-19 vaccines: Structure and stability. Int. J. Pharm..

[121] Kalluri R., LeBleu V.S. (2020). The biology, function, and biomedical applications of exosomes. Science.

[122] Amiri A., Bagherifar R., Ansari Dezfouli E., Kiaie S.H., Jafari R., Ramezani R. (2022). Exosomes as bio-inspired nanocarriers for RNA delivery: Preparation and applications. J. Transl. Med..

[123] Fu Z., Zhang X., Zhou X., Ur-Rehman U., Yu M., Liang H., Guo H., Guo X., Kong Y., Su Y. (2021). In vivo self-assembled small RNAs as a new generation of RNAi therapeutics. Cell Res..

[124] Xiao X., Wang X., Wang Y., Yu T., Huang L., Chen L., Li J., Zhang C., Zhang Y. (2018). Multi-Functional Peptide-MicroRNA Nanocomplex for Targeted MicroRNA Delivery and Function Imaging. Chemistry.

[125] Xiao X., Wang X., Gao H., Chen X., Li J., Zhang Y. (2018). Cell-Selective Delivery of MicroRNA with a MicroRNA-Peptide Conjugate Nanocomplex. Chem. Asian J..

[126] Wang X., Xiao X., Zhang B., Li J., Zhang Y. (2019). A self-assembled peptide nucleic acid-microRNA nanocomplex for dual modulation of cancer-related microRNAs. Chem. Commun..

[127] Wang J., Chen G., Liu N., Han X., Zhao F., Zhang L., Chen P. (2022). Strategies for improving the safety and RNAi efficacy of noncovalent peptide/siRNA nanocomplexes. Adv. Colloid Interface Sci..

[128] Li J., Mo L., Lu C.H., Fu T., Yang H.H., Tan W. (2016). Functional nucleic acid-based hydrogels for bioanalytical and biomedical applications. Chem. Soc. Rev..

[129] Zhao B., Zhou B., Shi K., Zhang R., Dong C., Xie D., Tang L., Tian Y., Qian Z., Yang L. (2021). Sustained and targeted delivery of siRNA/DP7-C nanoparticles from injectable thermosensitive hydrogel for hepatocellular carcinoma therapy. Cancer Sci..

[130] Li J., Kooger R., He M., Xiao X., Zheng L., Zhang Y. (2014). A supramolecular hydrogel as a carrier to deliver microRNA into the encapsulated cells. Chem. Commun..

[131] Xiao X., Hu J., Wang X., Huang L., Chen Y., Wang W., Li J., Zhang Y. (2016). A dual-functional supramolecular hydrogel based on a spiropyran-galactose conjugate for target-mediated and light-controlled delivery of microRNA into cells. Chem. Commun..

[132] Huang X., Wu G., Liu C., Hua X., Tang Z., Xiao Y., Chen W., Zhou J., Kong N., Huang P. (2021). Intercalation-Driven Formation of siRNA Nanogels for Cancer Therapy. Nano Lett..

